# Effect of serum progesterone levels on hCG trigger day on pregnancy outcomes in GnRH antagonist cycles

**DOI:** 10.3389/fendo.2022.982830

**Published:** 2022-09-28

**Authors:** Junwei Zhang, Mingze Du, Yanli Wu, Zhancai Wei, Yichun Guan

**Affiliations:** The Reproductive Center, The Third Affiliated Hospital of Zhengzhou University, Zhengzhou, China

**Keywords:** progesterone, GnRH antagonist, live birth rate, clinical pregnancy rate, *in vitro* fertilization

## Abstract

**Objective:**

The present study analyzed the effect of hCG trigger day progesterone (P) levels on the live birth rate (LBR) in the gonadotropin-releasing hormone (GnRH) antagonist protocol.

**Materials and methods:**

This study was a single-center retrospective study. *In vitro* fertilization (IVF)/intracytoplasmic sperm injection (ICSI) cycles performed from January 2017 to December 2020 were included in the analysis. This study included people with a normal ovarian response to fresh embryo transfer of GnRH antagonist protocols. All cycles were divided into 2 groups by P level on the day of human chorionic gonadotropin (hCG) trigger, P<1.0 ng/ml and P≥1.0 ng/ml. The primary outcome measure was LBR.

**Result:**

A total of 867 cycles with P<1.0 ng/ml and 362 cycles with P≥1.0 ng/ml were included in the analysis. The clinical pregnancy rate (CPR) was higher in the P<1.0 ng/ml group than the P≥1.0 ng/ml group (44.9% vs. 37.6%, P=0.02). The early spontaneous abortion rate was comparable between the groups (14.4% vs. 14.7%, P=0.93). For live birth, the rate for the P<1.0 ng/ml group was 35.3%, which was significantly higher than the 29.0% in the P≥1.0 ng/ml group (P=0.03). After binary logistic regression analysis, the P level on the hCG trigger day (adjusted odds ratio=0.74, 95% CI=0.55-0.99, P=0.04) was an independent risk factor for LBR. For the P level on the hCG trigger day, the LBR was lower in the P≥1.0 ng/ml group compared to the P<1.0 ng/ml group.

**Conclusion:**

For normal ovarian response patients using the GnRH antagonist protocol, serum P≥1.0 ng/ml on the hCG trigger day resulted in a lower LBR than the P<1.0 ng/ml group. When P≥1.0 ng/ml, whole embryo freezing may be considered.

## Introduction

Ovarian stimulation (OS) is a critical step for intracytoplasmic sperm injection (ICSI)/*in vitro* fertilization (IVF) (1). The rationale for OS is to achieve more follicle development using exogenous follicle-stimulating hormone (FSH), which stimulates the growth of multiple follicles in a single cycle (2, 3). However, the increase in estrogen (E2) caused by the development of multiple follicles increases luteinizing hormone (LH) levels before the follicles mature, which leads to earlier ovulation. Therefore, the key to OS is to prevent premature luteinization in advance. The most commonly used controls for endogenous LH peaks are gonadotropin-releasing hormone (GnRH) analogs, including GnRH agonists and GnRH antagonists (4, 5). A GnRH agonist protocol has been used in assisted reproductive technology since 1984 (6), and it is one of the most widely used OS protocols. GnRH agonists effectively inhibit the LH level and the occurrence of an early-onset LH surge, which improve the uniformity of follicle development. However, prolonged stimulation increases the gonadotropin (Gn) dose, which increases the risk of ovarian hyperstimulation syndrome (OHSS). GnRH antagonist protocols have been gradually used in the clinic since 2001. These protocols use a relatively short duration of stimulation with a lower Gn dose, which reduces the risk of OHSS (7, 8). Controversy exists in the use of GnRH agonists and GnRH antagonists (4, 9, 10). Based on the safety of OS, the GnRH antagonist protocol is more recommended for normal or high ovarian responders (1). However, the GnRH agonist protocol was more advantageous based on the live birth rate (LBR) of fresh embryo transfer, but there was no difference in the cumulative LBR between the two protocols (11).

Optimization of the LBR of fresh embryo transfer with GnRH antagonist protocols is the focus of much research. Serum progesterone (P) level is an important indicator in the pregnancy rate of the fresh cycle, and elevated P levels on the day of human chorionic gonadotropin (hCG) administration negatively influence clinical outcomes (12–14). Many studies examined the effect of serum P on clinical outcomes by measuring serum P levels on the day of the hCG trigger. The main reason for the controversy is that the threshold value of the serum P level is different between studies and ranges from 0.8 to 2.0 ng/ml, and there are differences in the determination methods (12, 15–17). The mechanisms of elevated P primarily include increased doses of gonadotropins (Gn), higher FSH levels, higher oocyte retrieval numbers and higher E2 levels on the trigger day (18). Therefore, the effect of elevated P levels on clinical outcomes may vary in different ovarian responders. Due to differences in populations, races, protocols, etc., the currently reported elevated P values are not uniform (13, 19, 20).

People with a normal ovarian response have a low risk of OHSS and a relatively stable number of oocytes retrieved and available embryos are the main population for fresh embryo transfer. Therefore, the present study analyzed the effect of P levels on hCG trigger day on LBR in a population with a normal ovarian response in a GnRH antagonist protocol.

## Methods

This study was a single-center, retrospective, observational, cohort study. This study was performed in the Reproductive Center of the Third Affiliated Hospital of Zhengzhou University. Ethical approval was obtained from the Ethics Committee of Third Affiliated Hospital of Zhengzhou University. IVF/ICSI cycles performed from January 2017 to December 2020 were included in the analysis.

### Population

A total of 1229 cycles of the GnRH antagonist protocol were included in the study analysis, all of which underwent the first IVF/ICSI cycle with fresh embryo transfer. This study included people with a normal ovarian response (age: 20-40 years old, baseline FSH<10 IU/L, anti-Mullerian hormone (AMH)≥1.1 ng/ml, antral follicle count (AFC)≥6). Patients with polycystic ovary syndrome were excluded from the analysis. Women with a history of uterine malformation (e.g., bicornuate uterus, unicornuate uterus or septate uterus), hydrosalpinx, history of ovarian surgery, adenomyosis or intrauterine adhesion were excluded from the analysis. Patients with recurrent spontaneous abortion were also excluded. All of the couples were screened *via* karyotyping, and couples with an abnormal karyotype were excluded.

### GnRH antagonist protocol and IVF/ICSI-embryo transfer

A routine flexible GnRH antagonist protocol was performed in our reproductive center as described in previous studies (21). OS was initiated on the second or third day of the menstrual cycle, and the appropriate Gn starting dose (100-300 IU) was chosen based on maternal age, weight, body mass index (BMI) and AMH. Vaginal ultrasonography was performed and serum LH and E2 levels were determined 3-5 days later. A GnRH antagonist (0.25 mg/day) was added once the diameter of the dominant follicle reached 12-14 mm and was continued up to the trigger day. The GnRH antagonist was injected at approximately 5 pm each day. If the LH peak occurred during the process of ovarian stimulation, the GnRH antagonist was also injected in time. When there were 3 follicles > 17 mm or 2 follicles > 18 mm, and patients were undergoing fresh embryo transfer, 250 μg recombinant hCG was applied for follicle maturation. Oocyte retrieval was performed 36 hours later. Based on sperm quality, conventional IVF or ICSI was performed, as appropriate. Luteal support was started on the day of oocyte retrieval using oral dydrogesterone (DYG; 10 mg, 2 times daily) (Abbott Co. America). Intravaginal progesterone sustained-release vaginal gel (90 mg, Merck Co. Germany) was given. One or two cleavage stage embryos were transferred 3 days after oocyte retrieval, or 1 blastocyst was transferred 5 days after oocyte retrieval. If pregnancy occurred, corpus luteum support was continued for at least 55 days after embryo transfer.

### Serum hormone level measurement and grouping

Serum hormone levels, including FSH, LH, E2 and P, were analyzed using the Roche Cobas immunoassay (Roche Diagnostics, Germany). The preparation, setup, dilution, adjustment, assay and quality control procedures were performed according to the manufacturer’s instructions, and the intra-assay and inter-assay coefficients of variation were less than 10%. On the hCG trigger day, whole blood was collected between 7:00 and 9 a.m. We routinely measured serum LH, E2 and P levels. Fresh embryo transfer was cancelled when the serum P level was >2 ng/ml, and whole embryo freezing was performed. We divided all cycles into 2 groups by the P level on the day of hCG trigger, P<1.0 ng/ml and P≥1.0 ng/ml. This grouping was primarily based on data distribution characteristics and reference to current related research (13, 22, 23).

### Outcome measures and definition

The primary outcome measure was LBR after fresh embryo transfer. Live birth was defined as any viable neonate ≥ 28 gestational weeks. The secondary outcome measure was clinical pregnancy rate (CPR), which was defined as a pregnancy diagnosed *via* ultrasonographic visualization of one or more gestational sacs and included intrauterine pregnancy and a clinically documented ectopic pregnancy (24). Early spontaneous abortion was defined as a loss of clinical pregnancy before 12 gestational weeks and was included as an outcome measure of this study.

### Statistical analysis

All data were obtained from retrospective review of our reproductive center’s medical records. All statistical management and analyses were performed using SPSS software, version 22.0.

For continuous variables, the one-sample Kolmogorov–Smirnov test was performed to check for normality. Continuous variables with abnormal distributions are expressed as medians (P25, P75), and the Wilcoxon rank sum test was used to assess between-group differences. Categorical variables are represented as the number of cases (n) and percentage (%). The between-group differences were assessed using chi-squared analyses with Fisher’s exact test when necessary. Binary logistic regression was performed to adjust for potential confounding factors for the main outcome, LBR. Adjusted odds ratios (AORs) with 95% confidence intervals (CIs) were calculated. Statistical significance was set at p value < 0.05.

## Results

### Study population

A total of 867 cycles with P<1.0 ng/ml and 362 cycles with P≥1.0 ng/ml were included for analysis. There were no statistically significant differences in maternal age, paternal age, duration of infertility, gravidity, type of infertility, infertility diagnosis, AMH, basal AFC or method of assisted reproductive technology (ART) (all p>0.05) between groups. The BMI in the P<1.0 ng/ml group was 23.8 (21.8, 26.0), which was significantly different than the P≥1.0 ng/ml group at 23.4 (21.4, 25.4) (p<0.01). Basal FSH was higher in the P<1.0 ng/ml group than the P≥1.0 ng/ml group (p<0.01). The detailed characteristics of the participants at baseline between the two groups are described in **Table 1**.

**Table 1:** Characteristics of the participants at baseline.

Characteristic	P<1.0 ng/ml	P≥1.0 ng/ml	p value
Number of cases	867	362	
Maternal age (year)	32.0 (29.0, 38.0)	33.0 (29.0, 38.0)	0.43
Paternal age (year)	33.0 (29.0, 38.0)	33.0 (30.0, 39.0)	0.41
Body mass index (kg/m2)	23.8 (21.8, 26.0)	23.4 (21.4, 25.4)	<0.01
Duration of infertility (year)	3.0 (2.0, 5.0)	3.0 (1.0, 5.0)	0.08
Gravidity	1 (0, 2)	1 (0, 2)	0.26
Type of infertility (%)			0.43
Primary infertility	38.2 (331/867)	40.6 (147/362)	
Secondary infertility	61.8 (536/867)	59.4 (215/362)	
Infertility diagnosis (%)			0.09
Tubal factor	33.8 (293/867)	32.6 (118/362)	
Male factor	14.9 (129/867)	20.7 (75/362)	
Male+female factors	22.6 (196/867)	19.9 (72/362)	
Others	28.7 (249/867)	26.8 (97/362)	
Basal FSH (IU/L)	7.1 (5.9, 8.8)	6.7 (5.6, 8.2)	<0.01
AMH (ng/ml)	1.9 (1.0, 4.5)	2.3 (1.3, 4.1)	0.10
Basal antral follicle count	12 (7, 16)	11 (8, 16)	0.95
Method of ART (%)			0.07
IVF	69.9 (606/867)	64.6 (234/362)	
ICSI	30.1 (261/867)	35.4 (128/362)	

Data are presented as medians (P25, P75) for continuous variables and % (n/N) for categorical variables.

### Characteristics of controlled ovarian hyperstimulation cycles

The starting dose of Gn, endometrial thickness on the hCG trigger day and type of transferred embryos (cleavage embryo/blastocyst) were comparable between the two groups (all p>0.05). There were statistically significant differences in the number of days of ovarian stimulation, total dose of Gn, estradiol level on hCG trigger day, number of follicles ≥14 mm, 16 mm and 18 mm on hCG trigger day, oocytes retrieved, two distinct pronuclei (2PN), available embryos on day 3 and available embryo rate between the groups. The number of transferred embryos was higher in the P<1.0 ng/ml group than the P≥1.0 ng/ml group (p=0.04). The detailed characteristics of the cycles between the two groups are described in **Table 2**.

**Table 2:** Characteristics of controlled ovarian hyperstimulation cycles.

Characteristic	P<1.0 ng/ml	P≥1.0 ng/ml	p value
Number of cases	867	362	
Starting dose of Gn (IU)	225.0 (187.5, 300.0)	225.0 (200.0, 300.0)	0.45
Number of days of ovarian stimulation	9 (8, 10)	10 (9, 11)	<0.01
Total dose of Gn (IU)	2400.0 (1800.0, 3000.0)	2625.0 (2000.0, 3000.0)	<0.01
Estradiol level on hCG trigger day (pg/ml)	1817.4 (1208.0, 2658.9)	2546.8 (1826.8, 3703.1)	<0.01
Number of follicles ≥ 14 mm on hCG trigger day	6 (4, 9)	8 (6, 11)	<0.01
Number of follicles ≥ 16 mm on hCG trigger day	5 (3, 6)	6 (4, 8)	<0.01
Number of follicles ≥ 18 mm on hCG trigger day	3 (2, 4)	3 (2, 5)	<0.01
Number of oocytes retrieved	7 (5, 11)	10 (7, 13)	<0.01
Number of 2PN	4 (3, 8)	6 (4, 9)	<0.01
Number of available embryos on day 3	4 (2, 6)	4 (2, 7)	<0.01
Available embryo rate (%)	50.0 (37.5, 70.0)	50.0 (33.3, 64.5)	<0.01
Endometrial thickness on the hCG trigger day (mm)	10.0 (8.8, 11.7)	10.0 (8.4, 12.0)	0.65
No. of transferred embryos			0.04
1	33.3 (289/867)	27.3 (99/362)	
2	66.7 (578/867)	72.7 (263/362)	
Type of transferred embryos			0.14
Cleavage embryo	86.2 (747/867)	82.9 (300/362)	
Blastocyst	13.8 (120/867)	17.1 (62/362)	

Data are presented as medians (P25, P75) for continuous variable and % (n/N) for categorical variables.

### Clinical outcomes

The CPR was higher in the P<1.0 ng/ml group than the P≥1.0 ng/ml group (44.9% vs. 37.6%, p=0.02). The early spontaneous abortion rate was comparable between the groups (14.4% vs. 14.7%, p=0.93). The LBR of the P<1.0 ng/ml group was 35.3%, which was significantly higher than the 29.0% in the P≥1.0 ng/ml group (p=0.03). A binary logistic regression model was performed to adjust the influence of confounding factors, including maternal age (continuous variable), maternal BMI (continuous variable), type of infertility (primary/secondary), infertility diagnosis (tubal/male/male+female/others), basal AFC (continuous variable), basal FSH (continuous variable), method of ART (IVF/ICSI), total dose of Gn, endometrial thickness on the hCG trigger day (continuous variable), number of oocytes retrieved (continuous variable), number of transferred embryos (1/2), type of transferred embryos (cleavage embryo/blastocyst) and P level on hCG trigger day (<1.0 ng/ml/≥1.0 ng/ml). Binary logistic regression analysis revealed that maternal age (AOR=0.91, 95% CI=0.88-0.93, p<0.01), endometrial thickness on the hCG trigger day (AOR=1.13, 95% CI=1.06-1.20, p<0.01), P level on hCG trigger day (AOR=0.74, 95% CI=0.55-0.99, p=0.04), number of transferred embryos (AOR=2.01, 95% CI=1.37-2.95, p<0.01) and type of transferred embryos (AOR=2.20, 95% CI=1.28-3.79, p<0.01) were independent risk factors for LBR. For the P level on the hCG trigger day, the LBR was lower in the P≥1.0 ng/ml group compared to the P<1 ng/ml group. The specific data are described in **Tables 3**, **4**.

**Table 3:** Clinical outcomes between the two groups.

	P<1.0 ng/ml	P≥1.0 ng/ml	p value
Clinical pregnancy rate (%)	44.9 (389/867)	37.6 (136/362)	0.02
Early spontaneous abortion rate (%)	14.4 (56/389)	14.7 (20/136)	0.93
Live birth rate (%)	35.3 (306/867)	29.0 (105/362)	0.03

Data are presented as % (n/N) for categorical variables.

**Table 4:** Binary logistic regression analysis to account for confounding variables of live birth rate.

	AOR	95%CI	p value
Maternal age (year)	0.91	0.88-0.93	<0.01
Body mass index (kg/m2)	0.99	0.95-1.04	0.77
Type of infertility (primary/secondary)	1.15	0.86-1.52	0.34
Infertility diagnosis (tubal/male/male+female/others)	0.96	0.86-1.06	0.42
Basal antral follicle count	1.01	0.99-1.04	0.23
Basal FSH (IU/L)	0.98	0.93-1.03	0.34
Method of ART (IVF/ICSI)	0.90	0.69-1.19	0.47
Total dose of Gn (IU)	1.00	1.00-1.00	0.15
Endometrial thickness on the hCG trigger day (mm)	1.13	1.06-1.20	<0.01
Number of oocytes retrieved	0.97	0.94-1.01	0.10
Progesterone level on hCG trigger day (<1/≥1.0 ng/ml)	0.74	0.55-0.99	0.04
Number of transferred embryos (1/2)	2.01	1.37-2.95	<0.01
Type of transferred embryos (cleavage embryo/blastocyst)	2.20	1.28-3.79	<0.01

AOR, adjusted odds ratio; CI, confidence interval.

## Discussion

Our single-center, retrospective cohort study involving normal ovarian response patients with the GnRH antagonist protocol found that the LBR for patients with P≥1.0 ng/ml was lower than patients with P<1.0 ng/ml. The risk of early spontaneous abortion did not differ significantly between the two groups.

There are many studies on the effects of P level on pregnancy. The synergistic effect of P and E2 is a necessary factor for embryo implantation in the natural state. Under the physiological state, when the dominant follicle is close to maturity and before ovulation, the follicle slightly increases the secretion of P to coordinate the positive feedback effect of E2 and induce the appearance of the peak of FSH and LH during ovulation. A large amount of P is secreted after ovulation (25). The increase in P level completely changes the endometrial state and endometrial receptivity (26). With the application of GnRH agonists and antagonists in OS cycles, the occurrence of endogenous LH peaks may be effectively prevented, but some patients still exhibit elevated serum P levels in the late follicular development stage (27). Schoolcraft et al. (28) first reported the phenomenon of elevated serum P levels on the day of hCG injection in some populations during the IVF treatment cycle with GnRH agonist protocols.

Subsequent reports of elevated P gradually appeared, with the overall incidence ranging from 5% to 38%. The incidence in GnRH agonist protocols ranged from 5% to 35%, and the incidence in GnRH antagonist protocols ranged from 9% to 38% (29, 30). Due to differences in study populations, laboratory testing methods, and groupings, the relationship between elevated serum P levels and IVF pregnancy outcomes remains controversial, and there are differences in the definition of elevated P. A large retrospective cohort study by Xu et al. (12) included populations with different ovarian responses and defined different serum P levels on the hCG trigger day. The defined values of serum P in patients with low ovarian response, normal response and high response were 1.5 ng/ml, 1.75 ng/ml and 2.25 ng/ml, respectively. Bosch et al. (31) and Van Vaerenbergh et al. (32) set the limit of the hCG daily P level to 1.5 ng/ml, which is widely used in clinical practice. However, studies show that when P>1 ng/ml, the CPR or LBR decreases (13, 19, 23). Only people with normal ovarian response with GnRH antagonist protocols were included for analysis in our study, and P>1 ng/ml affected the LBR of fresh embryo transfer. Fresh embryo transfer should be canceled, and whole embryo freezing should be performed when P is high.

Although many clinical studies showed that elevated serum P levels had a negative impact on CPR and LBR, the specific endocrine mechanisms are not clear. Major mechanistic studies focused on the effects of elevated serum P on endometrial receptivity and oocyte and embryo quality. Elevated serum P levels reduced CPR in fresh embryo transfer cycles but did not affect clinical outcomes in frozen-thawed embryo transfer cycles (33). Chen et al. (34) showed that high serum P does not affect embryo quality, and most studies believe that elevated serum P levels had no effect on oocyte quality, fertilization rate, or embryo quality. Santos-Ribeiro et al. (35) reported that IVF fertilization rates were similar between different P levels (≤0.50 ng/ml, 0.5–1.5 ng/ml and >1.5 ng/ml), which confirmed that serum P levels did not affect IVF fertilization rates. A recent study using the 90th percentile of the distribution of serum P levels as a basis for grouping also showed that P levels had no negative effects on oocyte or embryo quality (36). Embryo implantation theory suggests a specific implantation window for embryo implantation. When the endometrium and embryonic development are out of sync for more than 3 days, the pregnancy rate is extremely low (37). Premature elevation of serum P levels affects endometrial receptivity by altering the expression of endometrial-specific genes and promoting endometrial transition from early secretory to late secretory (38). The increase in serum P levels has a specific effect on the gene expression profile of the endometrium (32). Different serum P levels induce different gene expression in the endometrium, and the expression of specific genes is related to embryo adhesion, the implantation process, and the immune system, which affect CPR in fresh cycles (39).

According to the previous data of the center and other studies, fresh embryo transfer was cancelled when the serum P level on hCG day >2 ng/ml, and whole embryo freezing was performed in our reproductive center. However, current data at our center show that the LBR of fresh embryo transfer with GnRH antagonist remains lower than a GnRH agonist. Therefore, the present study investigated whether slightly elevated P levels also affected LBR. First, this study only included GnRH antagonist regimens with normal ovarian response to minimize the influence of confounding factors. Second, the observational endpoint of this study was the LBR, which is more clinically valuable than comparisons of only the CPR. However, the current study is limited by its retrospective cohort nature. Second, this study did not further analyze the impact of the quality of different embryos or blastocysts transferred on clinical outcomes, and there may be confounding factors. A retrospective cohort study revealed that a slight increase in P levels (0.85 ng/mL) affected the CPR of cleavage-stage embryo transfers, but it did not affect clinical outcomes after blastocyst transfer (40). Therefore, for patients with mildly elevated P, whether blastocyst transfer improves clinical outcomes is a direction of further research. The current study only included people with normal ovarian response, and further research is needed in populations with high and low ovarian responses.

## Conclusion

For normal ovarian response patients with the GnRH antagonist protocol, serum P≥1.0 ng/ml on the hCG trigger day resulted in a lower LBR compared to P<1.0 ng/ml. When serum P≥1.0, whole embryo freezing may be considered, followed by frozen-thawed embryo transfer.

## Data availability statement

The original contributions presented in the study are included in the article/supplementary material. Further inquiries can be directed to the corresponding author.

## Ethics statement

This study was approved by the Ethics Committee of the Third Affiliated Hospital of Zhengzhou University. Study reference number: 2022-198-01. Written informed consent for participation was not required for this study in accordance with the national legislation and the institutional requirements.

## Author contributions

JZ, MD and YG designed the study and selected the population to be included and excluded. YW and ZW were involved in the data extraction and analyses. MD reviewed the data. JZ was involved in drafting this article. All authors approved the final version of the manuscript. JZ and MD contributed equally to this article. All authors contributed to the article and approved the submitted version.

## Funding

The study was supported by 2021 Henan Province Medical Science and Technology Research and Joint Construction Project (LHGJ20210441, LHGJ20210451).

## Acknowledgments

We thank the patients who participated in the study and cooperated with the follow-up. We thank the medical staff at our reproductive center who participated in data entry and follow-up. We also thank American Journal Experts for their professional manuscript editing services.

## Conflict of interest

The authors declare that the research was conducted in the absence of any commercial or financial relationships that could be construed as a potential conflict of interest.

## Publisher’s note

All claims expressed in this article are solely those of the authors and do not necessarily represent those of their affiliated organizations, or those of the publisher, the editors and the reviewers. Any product that may be evaluated in this article, or claim that may be made by its manufacturer, is not guaranteed or endorsed by the publisher.
